# Salivary Alpha-Amylase Reactivity in Breast Cancer Survivors

**DOI:** 10.3390/ijerph13040353

**Published:** 2016-03-23

**Authors:** Cynthia Wan, Marie-Ève Couture-Lalande, Tasha A. Narain, Sophie Lebel, Catherine Bielajew

**Affiliations:** 1 Faculty of Social Sciences, School of Psychology, University of Ottawa, Ottawa, ON K1N 6N5, Canada; mcout103@uottawa.ca (M.-È.C.-L.); sophie.lebel@uottawa.ca (S.L.); catch@uottawa.ca (C.B.); 2 Department of Public Health Sciences, Faculty of Health Sciences, Queen’s University, Kingston, ON K7L 3N6, Canada; tasha.narain@queensu.ca

**Keywords:** alpha-amylase, stress, breast cancer survivorship

## Abstract

The two main components of the stress system are the hypothalamic-pituitary-adrenal (HPA) and sympathetic-adrenal-medullary (SAM) axes. While cortisol has been commonly used as a biomarker of HPA functioning, much less attention has been paid to the role of the SAM in this context. Studies have shown that long-term breast cancer survivors display abnormal reactive cortisol patterns, suggesting a dysregulation of their HPA axis. To fully understand the integrity of the stress response in this population, this paper explored the diurnal and acute alpha-amylase profiles of 22 breast cancer survivors and 26 women with no history of cancer. Results revealed that breast cancer survivors displayed identical but elevated patterns of alpha-amylase concentrations in both diurnal and acute profiles relative to that of healthy women, F (1, 39) = 17.95, *p* < 0.001 and F (1, 37) = 7.29, *p* = 0.010, respectively. The average area under the curve for the diurnal and reactive profiles was 631.54 ± 66.94 SEM and 1238.78 ± 111.84 SEM, respectively. This is in sharp contrast to their cortisol results, which showed normal diurnal and blunted acute patterns. The complexity of the stress system necessitates further investigation to understand the synergistic relationship of the HPA and SAM axes.

## 1. Introduction

It has been estimated that one in nine women will develop breast cancer during her lifetime [1]. In 2013, breast cancer accounted for 14% of all cancer deaths in Canadian women and it has been estimated that 24,400 women will be diagnosed with breast cancer in 2014 (approximately 26% of cancer cases in women) [1]. Now, due to improved screening and treatment procedures, the five-year survival rate of breast cancer in Canadian women has been steadily increasing and is now roughly 88%. Despite these positive numbers, the long-term emotional and physical stressors associated with being a cancer survivor can lead to serious health problems [2,3,4].

Stress is an influential factor in psychological and physical wellbeing. Breast cancer survivors, defined in this study as individuals with a diagnosis of breast cancer and currently in remission, may experience various stressors throughout the cancer trajectory. Stressors may be punctual—associated with the diagnosis, anxiety over the reactions of loved ones, or the treatment itself [5,6]; or chronic—persisting following treatment, due to fear of death or recurrence, or concerns with managing lifestyle changes, returning to work, and coping with the minutiae of everyday life [7,8].

The two main components of the stress system, the hypothalamic-pituitary-adrenal (HPA) and sympathetic-adrenal-medullary (SAM) axes, operate in tandem to maintain homeostasis and prepare the organism to deal with environmental challenges [9]. One way to assess their functioning is via the examination of the secretion patterns of specific stress-related biomarkers. The focus of this paper is the enzyme alpha-amylase, a stress biomarker of the sympathetic nervous system [10,11], and one that has received much less attention than the omnipresent cortisol in research on stress physiology. Cortisol, a steroid hormone secreted in response to stress, has a long history as a stress endocrine marker [12,13,14]. For that reason and comparison purposes, we first review background and data on cortisol.

### 1.1. Salivary Cortisol as a Stress Biomarker

Cortisol, one of a class of glucocorticoid hormones, is associated with the HPA axis and is an essential steroid hormone for regulating bodily functions [15]. Cortisol is released from the adrenal glands through a cascade of events initiated by a stressful stimulus, which includes interaction with a host of anatomical structures related to the sympathetic nervous system (SNS). Studies have shown that it has a relatively stable diurnal pattern in healthy individuals [14,16], with its peak typically occurring within 30 min to 60 min after waking and thereafter steadily declining throughout the day [16]. In the face of an acute stressor, cortisol secretion is briefly increased and usually returns to basal levels within one or two hours following stressor cessation [17].

It has been demonstrated that approximately 10% of the healthy population exhibits abnormal diurnal cortisol, also referred to as dysregulated cortisol patterns [14]. Such patterns are characterized by a significantly blunted diurnal cortisol response in comparison to that of healthy individuals [18,19,20]. In clinical populations, metastatic breast cancer survivors (stage 4), in particular, are much more likely to show abnormal diurnal cortisol rhythms [21].

Abercrombie *et al.* [18] noted that breast cancer survivors with greater metastatic severity showed higher mean cortisol levels and flatter diurnal cortisol rhythms. Moreover, Sephton *et al*. [22] showed that a blunted diurnal cortisol response, which they speculated to be associated with more rapid cancer progression, predicted higher mortality rates for metastatic breast cancer survivors [20]. In our laboratory, we have observed normal diurnal patterns in breast cancer survivors (mostly diagnosed with stage 2 or less), but a positive relationship between reactive salivary cortisol levels and time since diagnosis in breast cancer survivors; women diagnosed more than five years earlier show cortisol levels approaching that of control participants without a history of cancer [12].

### 1.2. Salivary Alpha-Amylase as a Stress Biomarker

Recently, investigators have become interested in exploring biomarkers that reflect SNS activity as a way of understanding the synergistic relationship between the HPA and SAM systems as adaptive stress mechanisms. Alpha-amylase, a salivary enzyme that is modulated by the autonomic nervous system (ANS), controls various visceral functions, such as salivation. Studies exploring the involvement of the ANS in the secretion of alpha-amylase first began in the early 1970s. In a series of studies examining the parotid gland in rats [23,24], Batzri’s group [23,24] noted that beta-adrenergic receptors were involved in the secretion of alpha-amylase. Subsequently, Anderson *et al.* [25] and Asking [26] evaluated the contributions of the parasympathetic and sympathetic branches of the ANS to alpha-amylase secretion in rats. They found that sympathetic stimulation of the parotid gland produced a low salivary flow rate with high concentrations of amylase, whereas the reverse was observed in response to parasympathetic stimulation [25,26]. Moreover, Asking [26] found that the effects of combined sympathetic and parasympathetic stimulation produced a synergistic effect and attributed the cause of greater alpha-amylase concentrations to beta-1-adrenoceptors.

While animal studies provided evidence for ANS involvement in alpha-amylase production, Speirs *et al*. [27] published one of the first studies to demonstrate the role of SNS in alpha-amylase production in humans and its connection to norepinephrine. In their study, participants were either immersed in cold water (4 °C to 5 °C) up to their waist or given isoprenaline (a beta-adrenergic agonist) or propranolol (a beta-adrenergic blocker) and found that the combined challenges increased alpha-amylase concentrations in the parotid gland while propranolol alone reduced it.

By the late 1970s, numerous researchers had begun to investigate the relationship between alpha-amylase and stress; the evidence generated since then suggests that alpha-amylase is a SNS biomarker and a reliable measure of both physical and psychological stress in humans [11,28,29,30,31]. Like cortisol, alpha-amylase exhibits a relatively stable diurnal pattern albeit distinctly different from that of cortisol [32]. Alpha-amylase concentrations are reduced significantly within 60 min after waking, followed by a gradual increase, with peak levels occurring in the late afternoon [32].

Alpha-amylase has been measured in response to a variety of acute stressors including writing a test [33], skydiving [34], and playing video games [35]. However, some studies have used a standardized psychosocial stress test such as the Trier Social Stress Test (TSST) to examine the reactivity of alpha-amylase and other biomarkers in response to psychologically induced stress.

The TSST is now considered the gold standard for inducing an acute stress response in the laboratory [36]. Although there may be some variations in the TSST from study to study, it typically has two components—a mock job interview followed by an arithmetic task [37]. The TSST has been shown to be very effective in producing stress-related physiological responses, including a two to threefold increase in salivary cortisol levels in 70% to 80% of participants, with peak levels occurring around 10 min to 20 min after TSST cessation [38]. Studies have reported significant alpha-amylase reactivity to the TSST in healthy children [28], youths [39], and adults [40].

The aim of the current study was to compare the circadian and reactive profiles of alpha-amylase in the same breast cancer survivors and in women with no history of breast cancer. Although researchers have begun examining alpha-amylase profiles in individuals with chronic illnesses [32], to our knowledge there is no study to date examining circadian and reactive profiles of alpha-amylase in breast cancer survivors. We reasoned that the stress associated with the breast cancer trajectory would be reflected by a dysregulation of the SNS, as indexed by salivary alpha amylase concentrations.

## 2. Method

### 2.1. Participants

A community sample of 22 female breast cancer survivors was recruited through printed advertisements and various cancer support groups. For complete participant demographics and medical characteristics, see Table 1 and Table 2.

Eligible participants were between the ages of 29 and 80 who understood English. Inclusion criteria for the breast cancer survivor group were as follows: A prior diagnosis of breast cancer (more than one year earlier) and completion of all local and/or systemic adjuvant therapy at least six months earlier. Individuals were ineligible if they had a previous history of other cancers (with the exception of non-invasive skin cancer and cervical cancer), substance abuse, or any major disabling conditions that would interfere with their quality of life. Women who were breast feeding or pregnant were also excluded from the current study. All eligible participants received $50 to compensate for any travel costs incurred and were entered into a draw for one of four $250 prizes. The study received ethical approval from the University of Ottawa Research Ethics Board (file number 04-09-04). All participants gave their written informed consent prior to inclusion in the study.

### 2.2. Measures of Stress

#### 2.2.1. Salivary Alpha-Amylase

Commercially available kinetic reaction assay kits were used to assay saliva samples for alpha-amylase, using protocols designed by Salimetrics, State College, PA, USA [41].

#### 2.2.2. Trier Social Stress Test (TSST)

The TSST [37] protocol we used consisted of two components—a mock job interview and an arithmetic task. Participants were given five minutes to prepare a five-minute free speech to a panel of three confederates acting as members of a hiring committee. This was followed by a five-minute arithmetic task that entailed the serial subtraction of 13 from 1022. In the event of an error, the participant was asked to start the task from the beginning. Committee members were instructed to provide no feedback to the participant.

#### 2.2.3. Visual Analog Scale (VAS)

A visual analog scale (VAS) [42] was used to measure subjective stress responses. It consisted of a 100 mm bipolar line on which participants were asked to estimate their stress level on a continuum from 0 equaling *not at all* to 100 equaling *very much* based on the statement “I feel stressed”. Scores were determined by measuring the distance from the left end to the appraisal mark.

#### 2.2.4. Questionnaires

Participants completed a series of questionnaires designed to provide socio-demographic information, and to assess their perception of stress. These included the Daily Stress Inventory [43], the Perceived Stress Scale [44], and the Life Experiences Survey [45]. Each measures a different aspect of stress and its impact on the individual. The Perceived Stress Scale is a 14-item questionnaire that measures subjective appraisals of situations as stressful in the past month, the Daily Stress Inventory of 58-items inquires about recent (past 24 h) stressful events and their intensity, and the Life Experiences Survey is a 57-item self-report questionnaire that measures the frequency and impact of positive and negative events that occurred in the past year.

Finally we also assessed the breast cancer survivors’ fear of recurrence using the Concerns About Recurrence Scale [46], a 30-item questionnaire. It evaluates five different domains related to fear of recurrence—worries pertaining to womanhood, role, health, death, and overall fear.

### 2.3. Procedure

Once participants were deemed eligible by telephone, they were scheduled at their convenience for two laboratory visits at the University of Ottawa. The first visit, approximately 30 min in length, served to obtain informed consent, and provide specific instructions on the correct method of collecting saliva samples at home. Afterwards, participants were given pre-labeled salivettes (synthetic swabs) to use at home at each time point—waking, 30 min after waking, 12:00 p.m., 4:00 p.m., and 9:00 p.m. They were instructed to rinse their mouths with water 10 min prior to collection (to avoid sample dilution), to place the salivette directly under their tongues for three minutes, and to store the salivettes in the refrigerator until delivery to the laboratory. Participants were also provided an insulated lunch bag with freezable ice packs to keep the samples cool in the event that they did not have access to a refrigerator and/or during their commute to the laboratory. They were also asked to avoid smoking and alcohol consumption 24 h before sample collection and eating or drinking caffeinated products and exercising one hour prior to sample collection. As a test of compliance, they entered the collection time in a recording book. Saliva samples were collected for two consecutive days. A second laboratory visit was scheduled within seven days following the home-based saliva collection.

**Figure 1:**
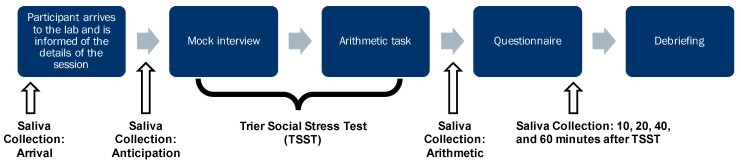
Schematic representation of the laboratory session procedure.

On the day of the visit, participants were asked to collect three saliva samples at home at waking, 30 min after waking, and at noon. The laboratory component lasted approximately two hours during which a total of seven saliva samples were collected; at each of the seven time points, participants were also asked to indicate their subjective stress level on the VAS. Figure 1 is a schematic illustration of the procedure of the laboratory session.

The first saliva sample, labeled “arrival”, was retrieved upon arrival to the laboratory. The participant was then escorted to a testing room and introduced to a mock panel of committee members and in their presence, explained the task instructions. These were to prepare a five-minute speech about her suitability for a mock job position and deliver it to the panel. No aids, such as notes, were permitted during the speech delivery. The arithmetic task immediately followed the speech presentation. Saliva samples were collected after the speech preparation (labeled “anticipation”) and, upon completion of the arithmetic task (labeled “arithmetic”).

Afterwards, the participant was asked to relax in the room for one hour and to complete a series of questionnaires assessing perceived stress, feelings of anxiety, and fear of recurrence. During this phase, four additional saliva samples were collected at 10 min, 20 min, 40 min, and 60 min. The participant was then debriefed and explained the purpose of the TSST. All saliva samples were transferred to Eppendorf tubes and stored in a freezer at −80 °C until assayed for alpha-amylase.

## 3. Results

### 3.1. Participant Characteristics

Details of the participant demographics and characteristics were reported in Couture-Lalande *et al.* [12]. Briefly, 22 breast cancer survivors and 26 women without a prior diagnosis of breast cancer completed the study. Participants were not matched, but both groups had a similar age distribution (average in late 50 s, t-test group difference *p* = 0.488), the number of women with postmenopausal status was similar (χ^2^ group difference *p* = 0.147), and roughly 90% self-identified as White. See Table 1 for details. Participants in the control group ranged from 29 years to 73 years of age, and participants in the breast cancer survivor group ranged from 39 years to 80 years of age (Table 1).

To our knowledge, there were no serious or untreated medical conditions in participants in either group. There were, however, a few cases of hypertension, diabetes (one or two per group), and osteoarthritis (in breast cancer survivors only); please refer to Couture-Lalande *et al.* [12] for more details. While studies suggest that the sympathetic nervous system may be implicated in the medical conditions mentioned, we chose to retain the participants’ data in order to avoid further reducing our sample sizes.

Table 2 provides the medical characteristics of the breast cancer survivors. The mean age at diagnosis was 54 ± 9 years (SD). On average participants were recruited about five years after diagnosis with the majority identified with stage 1 breast cancer. Most underwent surgical procedure for lumpectomy (approximately 45%). Two of the participants experienced a recurrence of breast cancer. Moreover, breast cancer survivors in the current study may have experienced one or a combination of the following treatments: chemotherapy, radiation, hormone therapy, and/or surgery. Please refer to Couture-Lalande *et al.* [12], for additional detail regarding the analysis of demographic and medical characteristics.

### 3.2. Data Analysis

Missing values were imputed using the EM algorithm in SPSS V22 (IBM Corporation, Armonk, NY, USA) for participants with up to two missing saliva samples of a total of 10 (usually due to inadequate saliva amounts) for their home-based saliva collection, and for participants with up to three missing samples out of the seven samples collected during the laboratory session. We imputed 4.6% of the diurnal and 5.7% of the TSST data related to alpha-amylase. For comparison to salivary cortisol, 3.8% and 5.7% were imputed for the same participants [12].

A series of mixed-design Analysis of Variance (ANOVA) were conducted to assess the diurnal and acute reactive profiles of alpha-amylase. In the event that Mauchly’s Test of Sphericity was significant, the Huynh–Feldt correction was applied, which adjusts the degrees of freedom [47].

### 3.3. Diurnal Alpha-Amylase

A 2 × 5 mixed-design Analysis of Variance (ANOVA) was used to assess differences in mean alpha-amylase concentrations over two consecutive days. The between-subject factor was *group* (breast cancer survivor or control) and the repeated factor was *time* (waking, 30 min after waking, 12:00 p.m., 4:00 p.m., and 9:00 p.m.).

**Figure 2:**
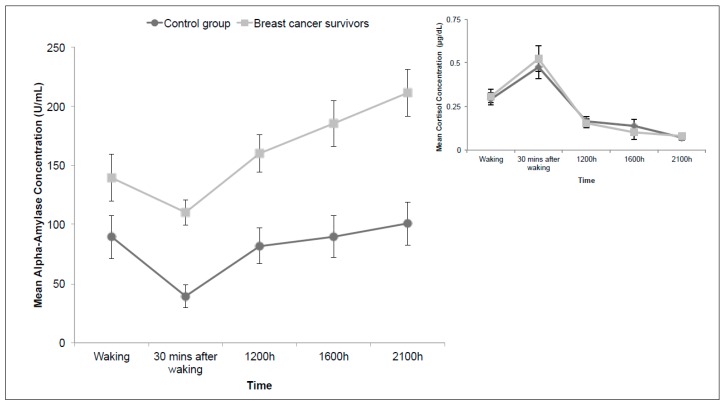
Mean diurnal alpha-amylase concentrations (U/mL) over two consecutive days. Inset graph includes published diurnal cortisol concentrations (ug/dL) for the same sample (Couture-Lalande *et al.* 2014). Error bars represent standard error of the mean.

Figure 2 shows the plot of the diurnal data. The inset graph on the upper right side contains the cortisol data from the same subjects for comparison purposes [12]. The statistical results revealed a significant main effect of time (F (3.39, 132.21) = 14.32, *p* < 0.001, η_p_^2^ = 0.269) and group (F (1, 39) = 17.95, *p* < 0.001, η_p_^2^ = 0.315) and no significant interaction between the two factors (*p* = 0.094). Across all time points, Bonferroni corrected main comparisons revealed that the mean alpha-amylase level at 30 min after waking was significantly lower than that associated with all other time points (*p* ranged from <0.001 to 0.038). Moreover, mean sAA levels were significantly lower at waking or shortly afterwards compared to that at the end of the day at 9:00 p.m. (*p* = 0.021).

As shown in Figure 2, although the group basal levels of alpha-amylase markedly different, the slopes of both patterns are similar; in contrast, the diurnal cortisol profiles that represent the two groups, shown in the inset graph, are virtually superimposable.

An additional analysis was performed to assess the relationship between time since diagnosis (in years) and diurnal alpha-amylase concentrations; no significant correlations were found at any of the five time points.

### 3.4. Alpha-Amylase in Response to Acute Stress

**Figure 3:**
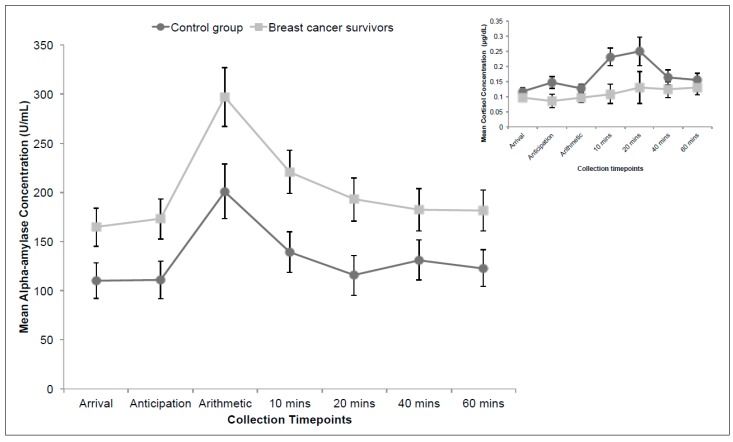
Mean acute alpha-amylase concentrations (U/mL). Inset graph includes published acute cortisol concentrations (ug/dL) for the same sample (Couture-Lalande *et al.* 2014). Error bars represent standard error of the mean.

Figure 3 depicts the salivary alpha-amylase profile in response to an acute stressor—the TSST. The inset graph shows the salivary cortisol data for the same subjects for comparison purposes (Couture-Lalande *et al.* 2014). A 2 × 7 mixed-design ANOVA was used to assess salivary alpha-amylase levels in this phase of the study. The between-group factor was *group* (breast cancer survivor or control participants) and the repeated factor was *time* (arrival, anticipation, arithmetic, and 10 min, 20 min, 40 min, and 60 min following the TSST).

The analysis revealed a significant main effect of time, F (4.75, 175.79) = 16.85, *p* < 0.001, η_p_^2^ = 0.313, and group, F (1, 37) = 7.29, *p* = 0.010, η_p_^2^ = 0.165, but no interaction (*p* = 0.563). Bonferroni corrected follow-up analysis revealed that sAA levels at Time 3 (“Arithmetic”) differed significantly from all other time points (*p* varied from <0.001 to 0.002). Furthermore, alpha-amylase concentrations did not differ significantly between Time 1 (Arrival) and Time 7 (60 min following the TSST), suggesting that all participants returned to baseline within one hour following the TSST (*p* = 1.00).

Similar to their sAA diurnal profile, breast cancer survivors had a higher basal level of alpha-amylase in comparison to the control group and a parallel time course, despite the difference in concentrations. This pattern contrasts with their acute cortisol patterns, shown in the inset graph of Figure 3; these were markedly different between the two groups with breast cancer survivors exhibiting a blunted cortisol response relative to that of the control group [12].

We also examined the correlation between time since diagnosis (in years) with alpha-amylase concentrations at all seven time points to assess whether sAA responses to an acute stressor were associated with the passage of time since diagnosis and found no significant relationships.

### 3.5. Area Under the Curve: Alpha-Amylase

Finally, we determined the area under the curve (auc) using trapezoidal integration for mean diurnal and reactive sAA levels in both groups. The auc for both groups was calculated with respect to the ground. The average auc for the diurnal and reactive sAA levels for breast cancer survivors was 631.54 ± 66.94 SEM and 1238.78 ± 111.84 SEM respectively. For diurnal and reactive sAA profiles in control participants, the values were 306.42 ± 39.23 SEM and 813.67 ± 107.02 SEM respectively. *t*-Test results revealed significant group differences in both the diurnal *t* (29.5) = −4.19, *p* < 0.001, *d* = 1.356, and reactive stress profiles *t* (37) = −2.74, *p* = 0.009, *d* = 0.88, due to consistently higher values in breast cancer survivors *versus* those of the control participants. Note that the group difference, averaged across all time points, was similar overall for both diurnal (36%) and reactive patterns (34%).

### 3.6. Subjective Measures in Relation to Alpha-Amylase

Results regarding subjective levels of stress were reported in a previously published study based on the same subjects; please see Couture-Lalande *et al.* [12] for an in-depth discussion of these analyses and results. Briefly, to document subjective levels of stress during the different phases of the TSST, participants were asked to indicate their overall stress at each time point, based on a visual analog scale. A mixed-design ANOVA was used to analyze these subjective responses; we found a significant main effect of time, *p* < 0.001, due to increased subjective stress during the anticipation, arithmetic, and 20 min time points, but there were no group or interaction effects. Indeed, the group patterns were virtually identical.

We also correlated each group’s scores from the three stress scales (Perceived Stress Scale, Daily Stress Inventory, and Life Experiences Survey) [43,44,45] with their average auc (area under the curve) for diurnal and reactive sAA levels and found no significant relationship.

Finally, for breast cancer survivors, we correlated their scores from the Concerns About Recurrence Scale (CARS) [43] with their average auc for diurnal and reactive sAA levels. Analyses revealed weak correlations between the various domains and average auc for diurnal and reactive sAA levels; diurnal data (*r values ranged from* 0.024 to 0.324 and *p values ranged from* 0.238 to 0.931); reactive data (*r values ranged from* 0.100 to 0.255 and *p values ranged from* 0.360 to 0.724).

## 4. Discussion

In a previous paper, we explored the diurnal and reactive patterns of cortisol in breast cancer survivors and women without a diagnosis of breast cancer [12]. Those data revealed almost identical group diurnal cortisol profiles and subjective appraisals of stress, but their cortisol levels in response to an acute stressor were considerably different. Breast cancer survivors exhibited a blunted cortisol response that showed some degree of normalization, that is, reactive cortisol patterns that approached that of the control group as a function of time since diagnosis [12].

The goal of the current study was to complement the cortisol findings by exploring diurnal stress and reactivity profiles based on sAA concentrations in the same participants. The breast cancer survivors varied from one to 11 years since diagnosis (*M* = 4.6 ± 3 years), but we did not find a correlation between “time since diagnosis” and cortisol or alpha-amylase concentrations at any of the collected time points. We reasoned that the apparent HPA dysregulation to an acute stressor in breast cancer survivors, as interpreted from cortisol patterns, would be accompanied by SNS impairments. To better understand the synergistic relationship between the HPA axis and SNS, it was therefore important to explore the diurnal and acute sAA profiles in the same individuals.

Based on our current sample, we found significant time and group differences in the diurnal and reactive alpha-amylase profiles of women with and without a prior diagnosis of cancer. These patterns are consistent with the literature, corroborating the findings of Nater *et al.* [48,49]. sAA concentrations decreased significantly immediately after waking, followed by a steady increase (see Figure 2). The reactive sAA patterns peaked at the arithmetic collection phase (see Figure 3). While both diurnal and reactive sAA patterns were elevated in breast cancer survivors, compared to that of women with no history of cancer, they increased to the same degree—36% increase in diurnal and 34% increase in reactive profiles. Thus the sAA difference in acute stress responses reflected a heightened basal level and was not related to a specific acute stress response. This is in contrast to cortisol group patterns, which showed a blunted response to the acute stressor in breast cancer survivors but otherwise diurnal rhythms identical to control participants.

Taken together, our data suggest that the experience of breast cancer is associated with dysregulated HPA and SAM functioning, possibly due to the various stressors that accompany a breast cancer diagnosis and its aftermath. The long-term state of chronic stress may have led to an allostatic overload [3,13]. *Allostasis* refers to the body’s active process of responding and adapting to external adverse events, in order to maintain homeostasis [50]. However, in the process of constant adaptation, problems such as allostatic overload may occur, which refers to the consequential biological wear and tear due to chronic stress. An allostatic overload may result in either an inadequate or overactive response or of the failure to adapt to repeated exposure to the same type of stressor [13,51].

To assess stress in the short and long term, we administered questionnaires that assessed levels of perceived stress in the past day, month, and over the last year, and found no significant relationship. For breast cancer survivors, we also added a questionnaire assessing their extent of fear of recurrence, a common chronic stressor experienced by most, if not all, cancer survivors, and likewise, found no association between particular worries and overall alpha-amylase values. While chronic stress may negatively impact the sympathetic nervous system, the levels observed in our sample did not appear to have any particular relationship to participants’ stress-related biomarker values.

Chronic stress has previously been documented to produce atypical cortisol levels (e.g., [12,52]. In a study investigating the functioning of HPA and SAM in response to repeated stress, Schommer *et al.* [53] exposed healthy participants to the TSST three times and found a mean decrease of 37% to 46% from TSST 1 to TSST 3 in HPA responses, whereas SNS responses remained unchanged. They concluded that the two systems respond to repeated psychosocial stress differently, and the HPA system, in particular, appears to habituate more quickly. They also noted that participants exhibited increased basal epinephrine levels at the third TSST, possibly due to the anticipation of stress.

Anticipation is an important aspect of allostasis [3]. Anticipation in this context refers to the psychological state of worry, anxiety, and cognitive preparation for an event [3]. By that definition, breast cancer survivors are in a constant state of anticipation. While the diagnosis and treatment of breast cancer is a stressful experience, anxiety, fears, and worries persist years after remission [54,55,56]. This state of chronic anxiety and stress may contribute to dysregulated HPA and SAM stress systems, but manifested differently in each case depending on its allostatic mechanism. Since breast cancer survivors are often distressed and anticipate adversity in the form of a recurrence or other cancer, it is plausible that this aspect disrupts stress-related SNS functioning by increasing alpha-amylase basal levels, representing an overactive response to adverse external events. In contrast, the HPA system via cortisol may signify an underactive response to chronic stress.

Associated with the notion of the accumulation of biological wear and tear is “age”, a determinant of alpha-amylase reactivity that has been shown to be associated with different sAA basal levels. It has been documented that basal levels of alpha-amylase reach that of adults’ values within the first three years and are then relatively stable across the life span [32]. However, some researchers suggest otherwise and have attributed attenuated sympathetic responses due to a chronic accumulation of sympathetic activation—the older the individual, the greater the SNS activation [10,57,58].

In a recent study investigating age differences in alpha-amylase reactivity, Almela *et al.* [59] had older adults (ages 54 to 71) and younger adults (ages 18 to 35) perform the TSST task or a control condition. In the latter, participants read aloud for five minutes followed by five minutes of counting. Almela *et al.* [59] did not find any group differences in sAA reactivity, but reported that overall sAA levels were higher in older adults. Moreover, they reported that in the stress condition, the total amount of cortisol secretion was positively related to the total amount of sAA secretion and heart rate increase was positively related to sAA increase. On that basis, they suggested that rather than an attenuated autonomic nervous system, a “heightened sympathetic tone” was a better explanation.

While it is possible that chronic stress and its consequent cumulative sympathetic activation with age may eventually lead to dysregulated stress systems in healthy individuals, chronic illnesses, such as a breast cancer diagnosis, may pose additional stress and exacerbate the effects. But these conclusions must be interpreted with caution; alpha-amylase has only recently been purported to be a surrogate SNS stress biomarker, and our understanding of its role in stress response and regulation is still expanding.

Only a scarce number of studies have explored diurnal sAA rhythms in sufferers of somatic or psychiatric diseases, or physical ailments, such as periodontitis [32], asthma [60], and posttraumatic stress disorder [61]. Several researchers have found lower sAA levels in pediatric patients suffering from a variety of chronic illnesses such as asthma and juvenile arthritis [32], but generally elevated sAA levels in different adult samples. For example, higher sAA concentrations were observed in adult patients with obstructive pulmonary disease, whereas lower sAA levels were found in adult patients who self-identified as habitual smokers [62]. Higher sAA levels were also recorded in individuals suffering from Parkinson’s disease [63] and type-2 diabetes [64].

Thoma *et al.* [61] recently published a study exploring the relationship between posttraumatic stress disorder and diurnal sAA patterns, and reported that the awakening response, in particular, was altered in comparison to that of healthy control participants. They found that sAA levels of patients suffering from posttraumatic stress disorder increased after waking, whereas sAA levels associated with the control group showed a sharp decrease in sAA levels after waking. Although limited, these findings point to the importance of evaluating other factors that appear to influence sAA profiles such as type of illness and age, and the individual’s role—for example, patient *versus* caregiver [65].

Briefly, Rohleder *et al.* [65] assessed the inflammatory and neurohormonal processes in caregivers over a one-year period. They noted that half the caregivers in the study showed a substantial linear increase in systemic inflammation, which if prolonged, may lead to low-grade chronic inflammation. Their endocrine results indicated that over time, caregivers’ diurnal secretion of sAA declined—that is, displaying a flattening of slope. However, their diurnal cortisol patterns were not affected.

Research concerning alpha-amylase’s role in stress reactivity in cancer patients and caregivers is still in its infancy. The complexity of the stress system and its interaction with chronic illness required further investigation to understand the synergistic relationship of the HPA and SAM axes in this context.

## 5. Limitations and Future Directions

The current study relied on convenience sampling and thus, may not be representative of all breast cancer survivors. Although the sample size is relatively small, it rivals the very few studies that have assessed sAA levels in breast cancer survivors—from about 20 per treatment group in an exploratory study [66] to over 100 in cross-sectional studies of breast cancer survivors [67].

Alpha-amylase levels can be affected by behavioural and life-style factors such as smoking, drinking, and physical exercise. Hormonal fluctuations due to menstrual cycle, the use of hormonal contraceptives, or hormonal replacement therapy also may impact alpha-amylase concentrations. In addition, the type of treatment received, such as chemotherapy and/or, radiation, certain chronic health conditions such as hypertension and diabetes, may be critical factors. Although we accounted for smoking and drinking habits in the study, we did not control for exercise habits or the use of hormonal contraception or hormonal replacement therapy. Future studies should investigate the influence of particular treatments and strive to include participants without other chronic pre-existing medical conditions. Our sample size was insufficient to perform a sub-group analysis based on treatment and menstrual status, but accounting for the nuances caused by hormonal fluctuations will be an important aspect to investigate.

Finally, the stress measures used in the current study evaluated primarily acute or short-term stress (within the last month, for example). The Life Experiences Survey only addressed the frequency and impact of perceived major stressful events that occurred in the past year; most participants did not indicate having such experiences. It would be important to explore in this context the influences of experiences over the lifetime and not limited to a particular time frame.

## 6. Conclusions

In the current study, we did not find atypical sAA profiles in breast cancer survivors. Rather, we found that breast cancer survivors exhibited elevated basal sAA levels, but the patterns paralleled the alpha-amylase reactivity of healthy women. The patterns were not related to the occurrence of stressful events in either group. More research is needed to determine the factors that influence the relationship between disease and stress biomarker patterns. Other routes could include the moderating effects of coping and social seeking behavior, fear of recurrence, and lifestyle habits. The relationship between HPA and SAM axes in maintaining homeostasis and managing stress is an important area of investigation, in order to understand their synergistic mechanisms and the consequences of their functioning in chronic disease.

## Figures and Tables

**Table 1:** Demographic characteristics of participants.

Demographic Characteristics	Breast Cancer Survivors (*N* = 22)	Control Group (*N* = 26)
Age (years) mean ± SD	58.9 ± 10.1	57.4 ± 11
	**No. of Participants (%)**	**No. of Participants (%)**
**Ethnicity**		
White	20 (90.9)	23 (88.5)
Black		1 (3.8)
Asian		2 (7.7)
First Nations	2 (9.1)	
**Highest level of education**		
High School	6 (27.3)	9 (34.6)
College	4 (18.2)	4 (15.4)
Bachelor’s degree	11 (50)	7 (26.9)
Master’s degree	1 (4.5)	5 (19.2)
Doctoral degree		1 (3.8)
Family income (CDN) *		
Under $40,000	3 (15)	5 (20.8)
$40,000 to $79,999	10 (50)	10 (41.7)
$80,000 to $ 119,999	5 (25)	5 (20.8)
$120,000 and over	2 (10)	4 (16.7)

* Breast cancer survivor group (*N* = 20); Control group (*N* = 24).

**Table 2:** Medical characteristics of breast cancer survivors.

Medical Characteristics	Breast Cancer Survivors (*N* = 22)
Mean age of diagnosis ± SD (years)	54.1 ± 8.7
Mean time (years) since diagnosis ± SD (years)	4.6 ± 3
**Breast cancer stage**	**No. of Participants (%)**
0	4 (18.2)
1	10 (45.5)
2	5 (22.7)
3	2 (13.6)
**Type of surgery**	
Unilateral mastectomy	6 (27.3)
Bilateral mastectomy	7 (31.8)
Lumpectomy	9 (40.9)
Treatment *	
Chemotherapy	10 (45.5)
Hormone therapy	14 (63.6)
Radiation therapy	14 (63.6)
**Breast cancer recurrence**	
None	20 (83.3)
One recurrence	1 (4.2)
Two recurrences	1 (4.2)

* Almost all participants received a combination of treatments.
